# Supplementary material for: A deterministic, c-di-GMP-dependent program ensures the generation of phenotypically similar, symmetric daughter cells during cytokinesis
Source: Nat Commun. 2024 Jul 17;15:6014. doi: 10.1038/s41467-024-50444-4 (PMC11255338; doi:10.1038/s41467-024-50444-4)
Supplement: Supplementary file 1 — Supplementary Information [file 41467_2024_50444_MOESM1_ESM.pdf]

Supplementary Information for

A deterministic, c-di-GMP-dependent program ensures the generation of phenotypically similar, symmetric daughter cells during cytokinesis

María Pérez-Burgos, Marco Herfurth, Andreas Kaczmarczyk, Andrea Harms, Katrin Huber, Urs Jenal, Timo Glatter, and Lotte Søgaard-Andersen

This file contains:

- Supplementary Figures 1-6
- Supplementary Table S1-S6
- Supplementary References

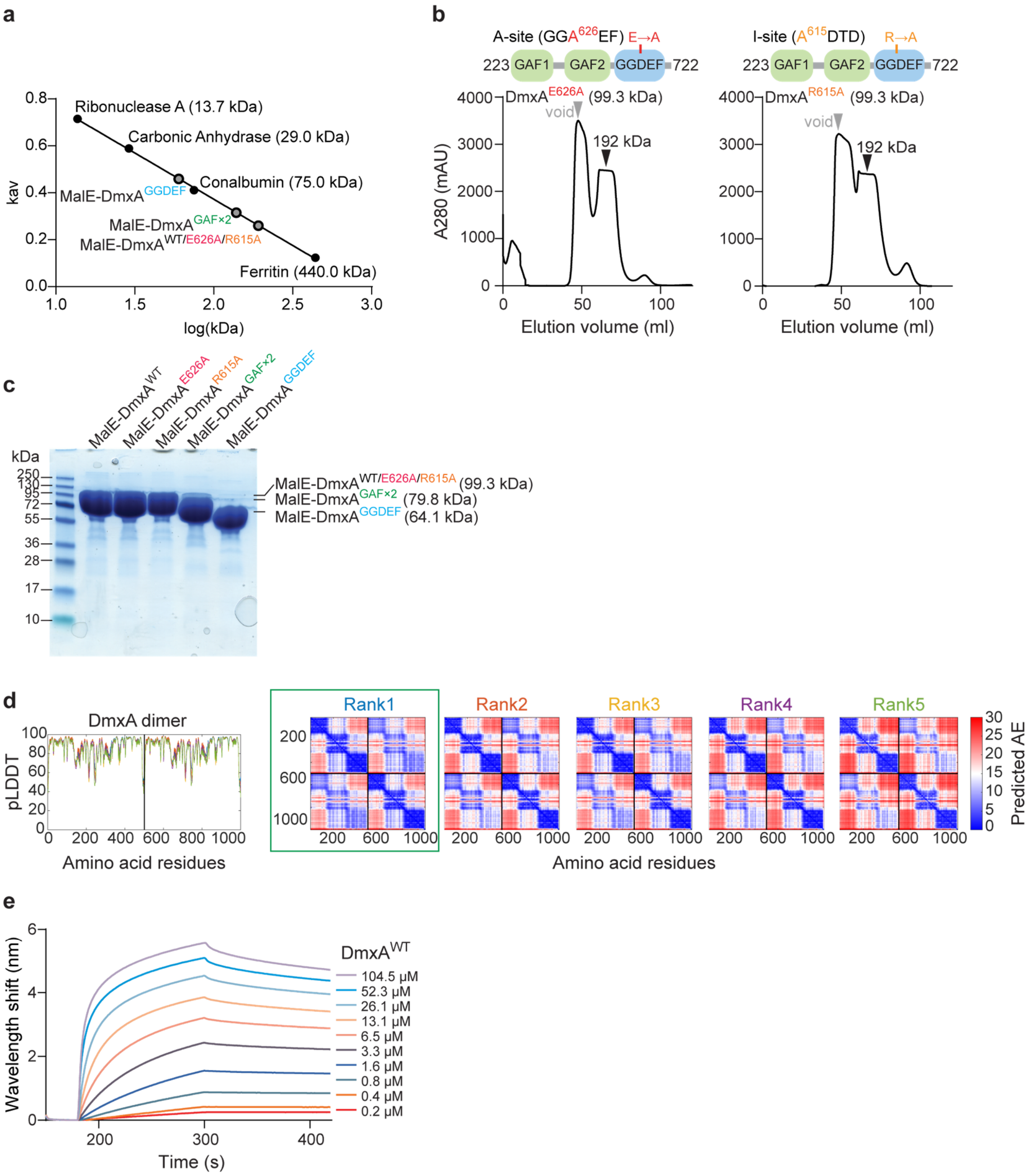

Figure S1. ***In silico*** **and** ***in vitro*** **analysis of DmxA variants.**

a, Calibration curve of SEC column. Molecular weight standards including their molecular mass are indicated in black and MalE-DmxA variants in grey dots. **b,** SEC of MalE-DmxA variants. Domain architectures of truncated DmxA variants are shown above chromatograms. Gray arrows indicate the void volume and black arrows the elution volume with the corresponding calculated molecular weight. **c**, Purified MalE-tagged DmxA variants after SEC. The MalE-tagged DmxA variants were separated by SDS-PAGE and stained with Coomassie blue. Calculated molecular weights of proteins are indicated. Molecular size markers are indicated on the left. **d**, pLDDT and pAE plots for five models of the DmxA dimer structure in Fig. 1c predicted by AlphaFold-Multimer. The model marked by a green box was

used for further analysis. **e**, Bio-Layer Interferometric analysis of the binding kinetics of MalE-tagged $DmxA^{WT}$ to c-di-GMP. Streptavidin-coated sensors were loaded with 500 nM biotinylated c-di-GMP and probed with indicated concentrations of MalE-$DmxA^{WT}$. The interaction kinetics were followed by monitoring the wavelength shifts during the association or dissociation of the analyte. Source data are provided as a Source Data file.

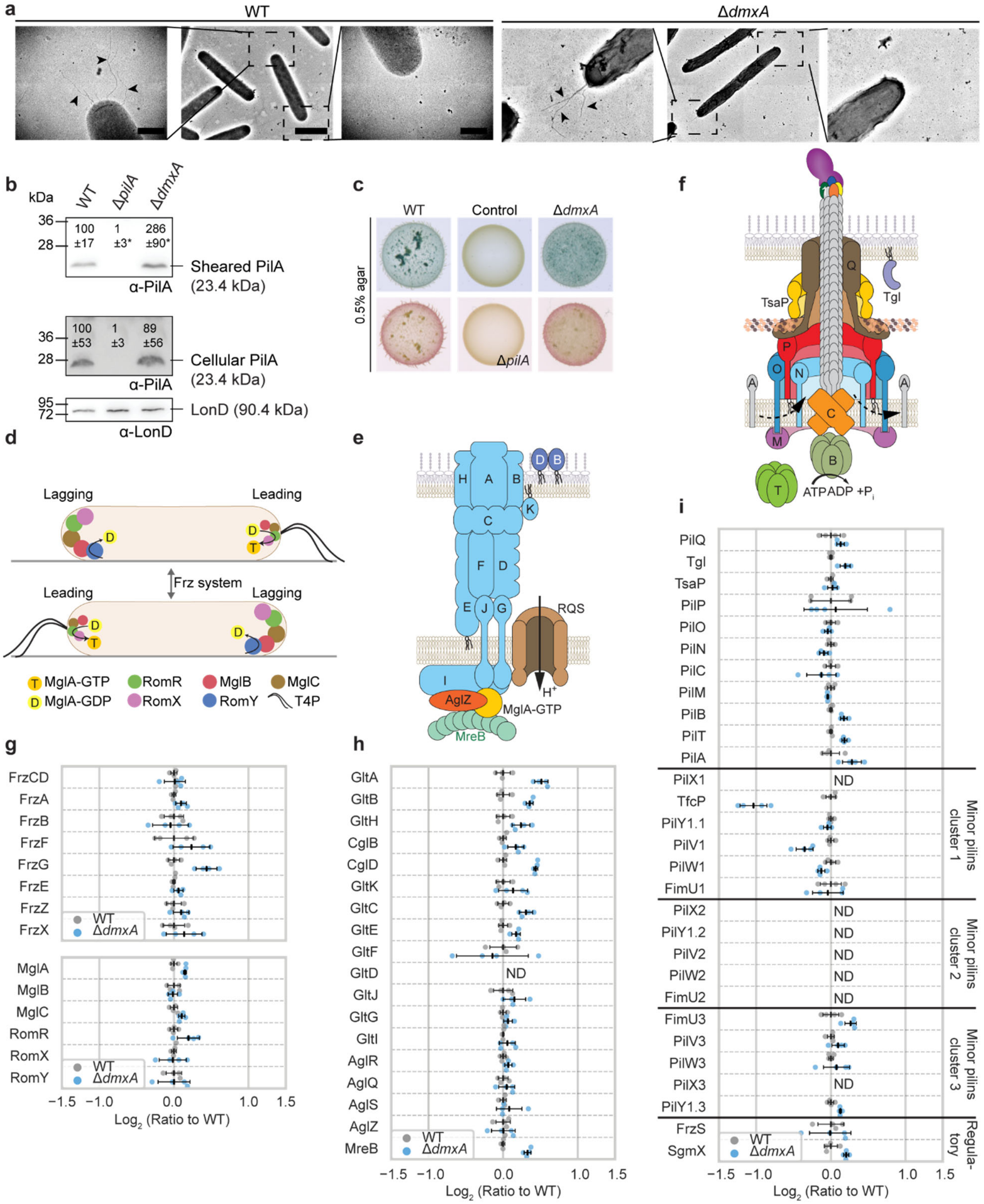

Figure S2. Phenotypic characterization and proteome analysis of the Δ*dmxA* mutant. **a**, Transmission electron microscopy of representative WT and a Δ*dmxA* cells with T4P at one pole. T4P are indicated by black arrowheads. The image in the middle shows the complete cell and the images on the left and right show the two poles. Scale bars, 0.5 µm (left and right), 2 µm (middle). **b,** T4P shear-off assay. Immunoblot detection of PilA in sheared T4P (top) and in total cell extract (bottom). Protein was isolated from the same number of cells from the indicated strains grown on 1% CTT 1.5% agar plates. Protein from the same number of cells was loaded per lane. The blot for cellular PilA was stripped and

then probed with α-LonD as a loading control. Calculated molecular weights of proteins are indicated. Numbers indicate the average level of PilA from n=3 biological replicates normalized to the loading control and relative to WT (100%). * p=0.0006 for Δ*pilA* vs WT; p=0.0245 for Δ*dmxA* vs WT, two-sided Student's t-test compared to WT. **c,** Determination of EPS synthesis. Cell suspensions of strains of the indicated genotypes were spotted on 0.5% agar supplemented with 0.5% CTT and Congo red or Trypan blue and incubated for 24 h. The Δ*pilA* mutant does not accumulate EPS[1] and was used as negative control. **d-f** Localization of the proteins of the polarity module (d) as well as schematics of the gliding motility complex (e) and the T4PM (f). In **d**, proteins of the polarity module are indicated[2-10]. Upon signaling by the Frz chemosensory system, their polarity is inverted. For each protein, the size of a circle indicates the amount of polarly localized protein. In **e**, proteins labeled with single letters in light blue, dark blue or brown have the Glt, Cgl or Agl prefix, respectively[11-18]. The AglR/-Q/-S complex harnesses proton-motive force to power gliding motility[19, 20]. In **f**, proteins labeled with single letters have the Pil prefix. The pilus fiber is formed by PilA subunits and a tip complex, composed of PilY1 and four minor pilins (blue: PilX, orange: PilV, green: PilW, yellow: FimU)[21, 22]. Tgl is an OM lipoprotein that is required for PilQ secretin assembly[23-25]. The ATPases PilB and PilT interact with the base of the T4PM in a mutually exclusive manner to stimulate extension and retraction of the pilus, respectively[21, 26, 27]. Bent arrows indicate incorporation of and removal of PilA subunits from the pilus base during extension and retraction, respectively. In **e** and **f**, lipoproteins are indicated by wavy black lines. **g-i**, Log_2 fold-change of the accumulation of proteins of the Frz chemosensory system and the polarity module (g), the Glt/Agl complex (h), and the T4PM (i) in the Δ*dmxA* strain compared to WT. ND, not detected. Data points represent n=4 biological replicates. Error bars, mean±SD based on these four replicates. Source data are provided as a Source Data file.

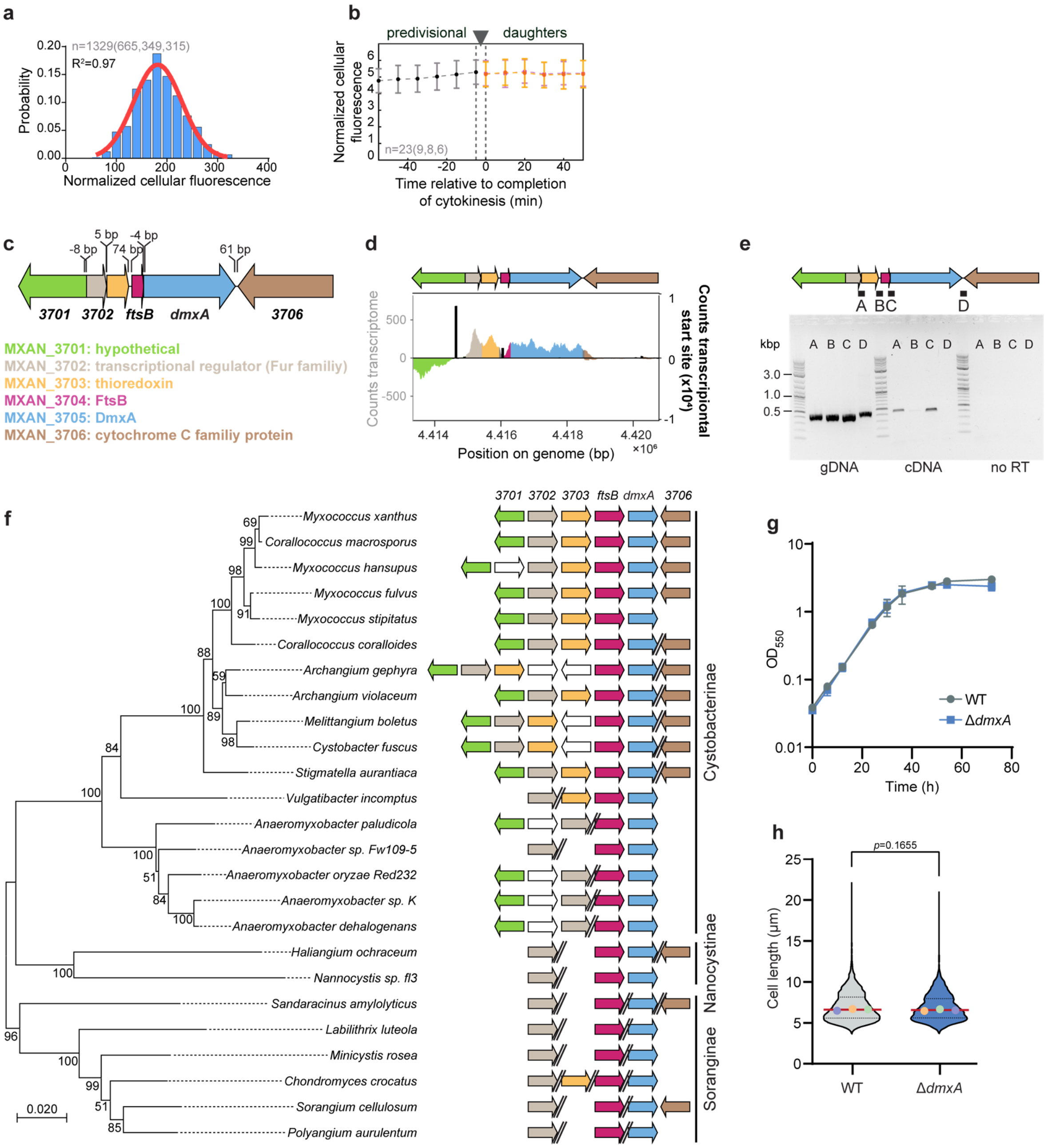

Figure S3. Analysis of DmxA-mVenus accumulation and the *dmxA* locus.
a, Distribution of the normalized cellular DmxA-mVenus fluorescence in single cells (n=1329 from three biological replicates, number of cells per replicate in brackets). Single cell total cellular fluorescence was normalized by the cell area to obtain the normalized cellular fluorescence. The distribution of the normalized cellular florescence is shown in histogram and was fitted to a Gaussian curve indicating a good fit to a unimodal distribution. **b,** Quantification of normalized cellular DmxA-mVenus fluorescence in time-lapse microscopy (n=23 division events from three biological replicates, number of events per replicate in brackets). DmxA-mVenus signals were determined before division in the predivisional cell (grey) and after division in the daughter cells (pink and yellow) at the indicated time points. Error bars, mean±SD. **c**, *dmxA* locus*.* Genes are drawn to scale and *MXAN* numbers or gene names are indicated. Predicted protein function is indicated. **d**, RNA-seq and

Cappable-seq as base-by-base alignment coverage for total RNA isolated from cells growing in 1% CTT broth[28]. Upper panel, genetic organization of *dmxA* locus as in c. Lower panel, positive and negative values indicate reads mapped to the forward and reverse strand, respectively. Reads assigned to a gene are colored according to the gene colour code in **c**; intergenic regions are in gray. Cappable-seq coverage is shown as black bars. **e**, Operon mapping of the *dmxA* locus. Upper panel, genetic organization of *dmxA* locus as in **c**. Letters and black lines below the genes indicate the fragments amplified by PCR (~400-500 bp). Bottom panel, the PCR products amplified using genomic DNA, cDNA, and an enzyme-free reverse transcription reaction (no RT) as templates were separated on a 1% agarose gel. Letters above the individual lanes correspond to the letters of the primer combinations depicted above. Molecular size markers in kilo base-pairs (kbp) are shown on the left. Representative image from n=2 biological replicates. **f**, Conservation of *dmxA* and its genetic neighborhood in other Myxococcales. Species included are listed in Table S6. Double slashes indicate no close proximity between the genes. **g**, Growth curve. Exponentially growing cells were diluted to an optical density (OD) at 550 nm (OD_{550}) of 0.04 and growth was followed over time. Growth curves were generated from n=3 biological replicates. Error bars, mean±SD. **h**, Cell length determination. The cell length distribution of three biological replicates is shown in a violin plot. Each violin indicates the probability density of the data at different cell length values. Single points represent the median of the three biological replicates indicated in different colours (n=4956 from three biological replicates with each 1652 cells). Median is represented by a continuous red line. Dashed lines indicate 25th and 75th percentiles. Samples were compared using a two-sided Mann-Whitney test. Source data are provided as a Source Data file.

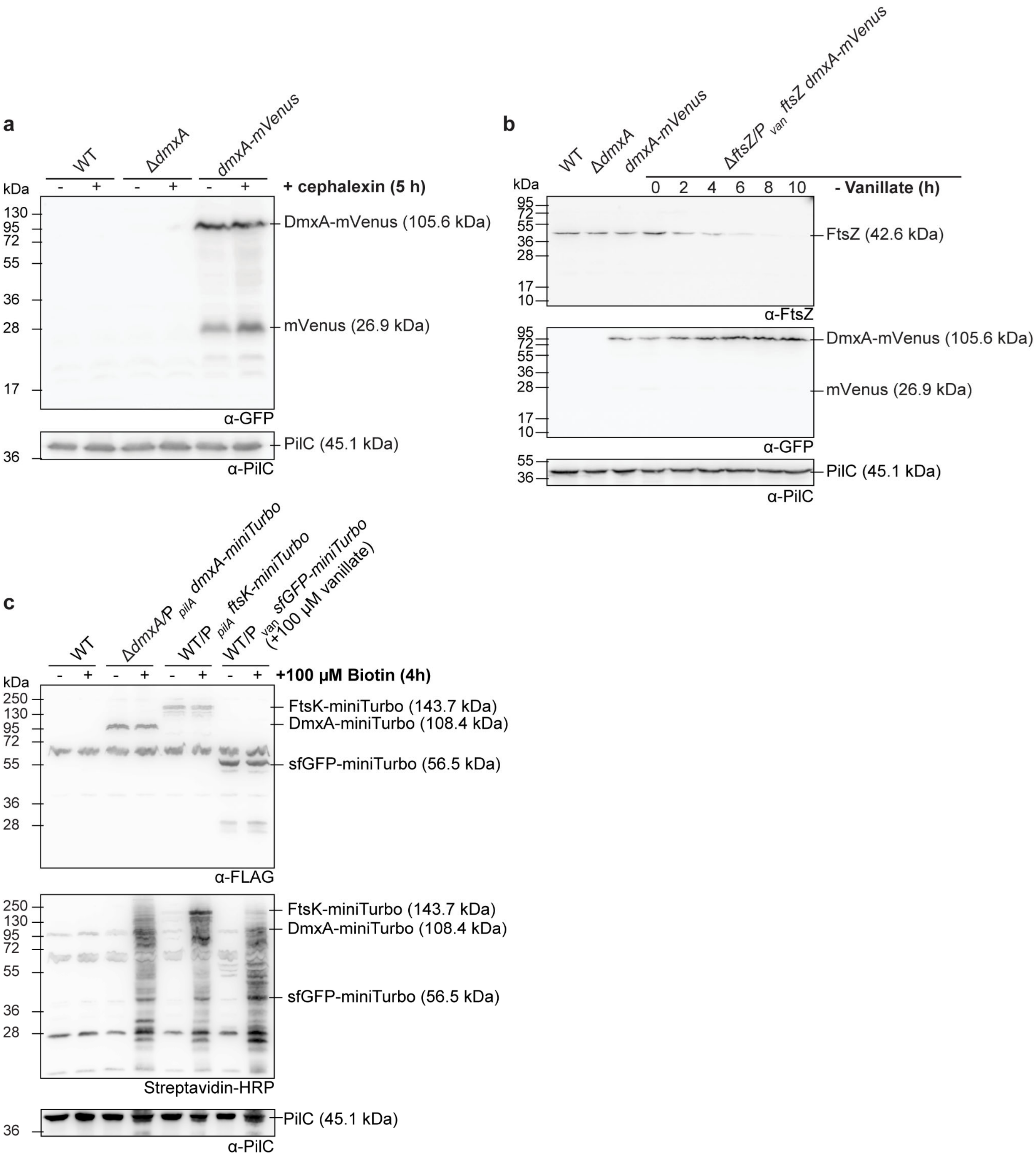

Figure S4. Accumulation of DmxA-mVenus, FtsZ and miniTurbo variants.

a, Immunoblot detection of DmxA-mVenus. Protein from 5.60×10^8 cells from exponentially growing suspension cultures treated with cephalexin for 5 h as indicated was loaded per lane. The same blot was stripped before applying a new antibody. PilC served as a loading control. Calculated molecular weights of proteins are indicated. **b**, Immunoblot detection of FtsZ and DmxA-mVenus. Protein from 1.05×10^8 cells from exponentially growing suspension cultures was loaded per lane. For the depletion of FtsZ, cells were exponentially grown in suspension culture in the presence of 10 µM vanillate for ~8 generations before the start of the experiment. Then, cells were washed and subsequently grown for 10 h without vanillate. The same blot was stripped before applying a new antibody. PilC served as a loading

control. Calculated molecular weights of proteins are indicated. **c**, Immunoblot detection of DmxA-, FtsK- and sfGFP-miniTurbo. DmxA-miniTurbo-FLAG and FtsK-miniTurbo-FLAG were expressed from the *pilA* promoter (P_{pilA}) and sfGFP-miniTurbo-FLAG from the P_{van} in the presence of 100 µM vanillate. Protein from 7.00×10^{7} cells from exponentially growing suspension cultures treated with or without biotin for 4 h was loaded per lane. The same blot was stripped before applying a new antibody. PilC served as a loading control. Western blots are representative from n=2 biological replicates. Source data are provided as a Source Data file.

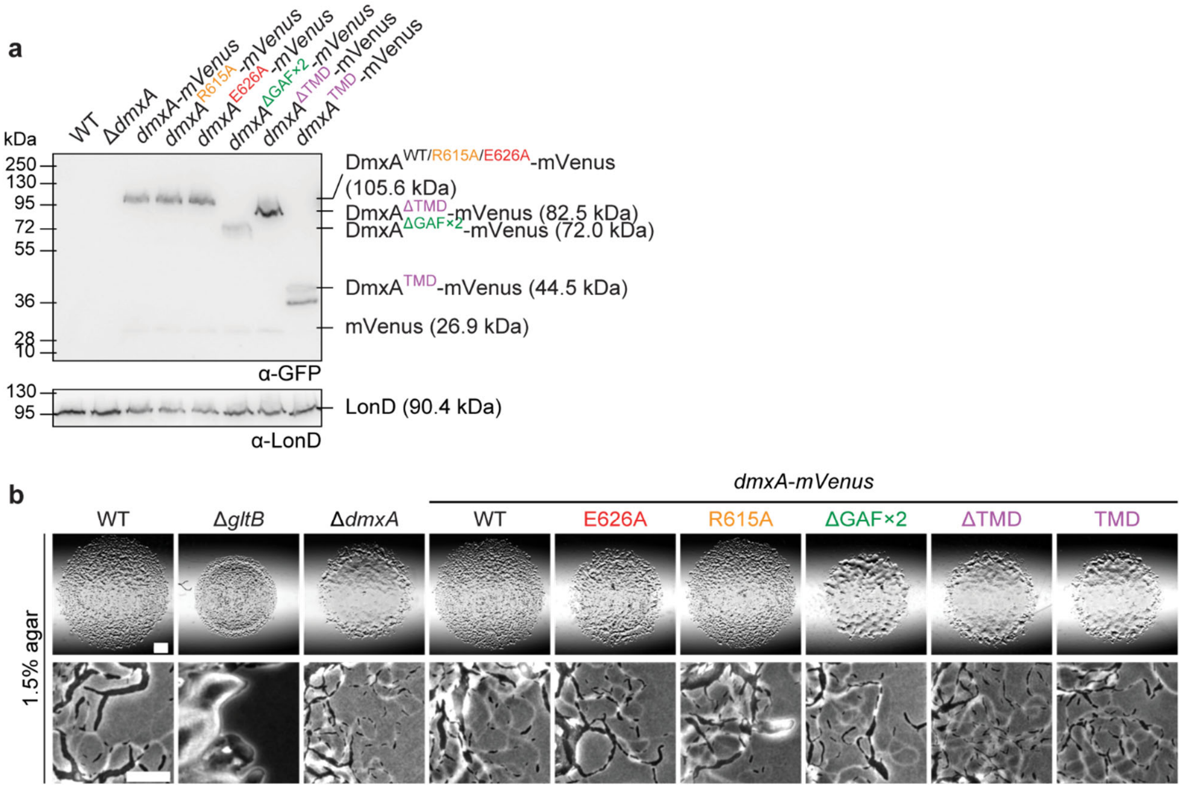

Figure S5. Accumulation of DmxA-mVenus variants and gliding in their presence.
a, Immunoblot detection of DmxA-mVenus variants. Protein from 1.40×10^{8} cells from exponentially growing suspension cultures was loaded per lane. The same blot was stripped before applying a new antibody. LonD served as a loading control. Calculated molecular weights of proteins are indicated. Representative blots from n=2 biological replicates. **b**, Gliding was analyzed on 1.5% agar. Scale bars, 1 mm (upper panels), 50 µm (lower panels). Source data are provided as a Source Data file.

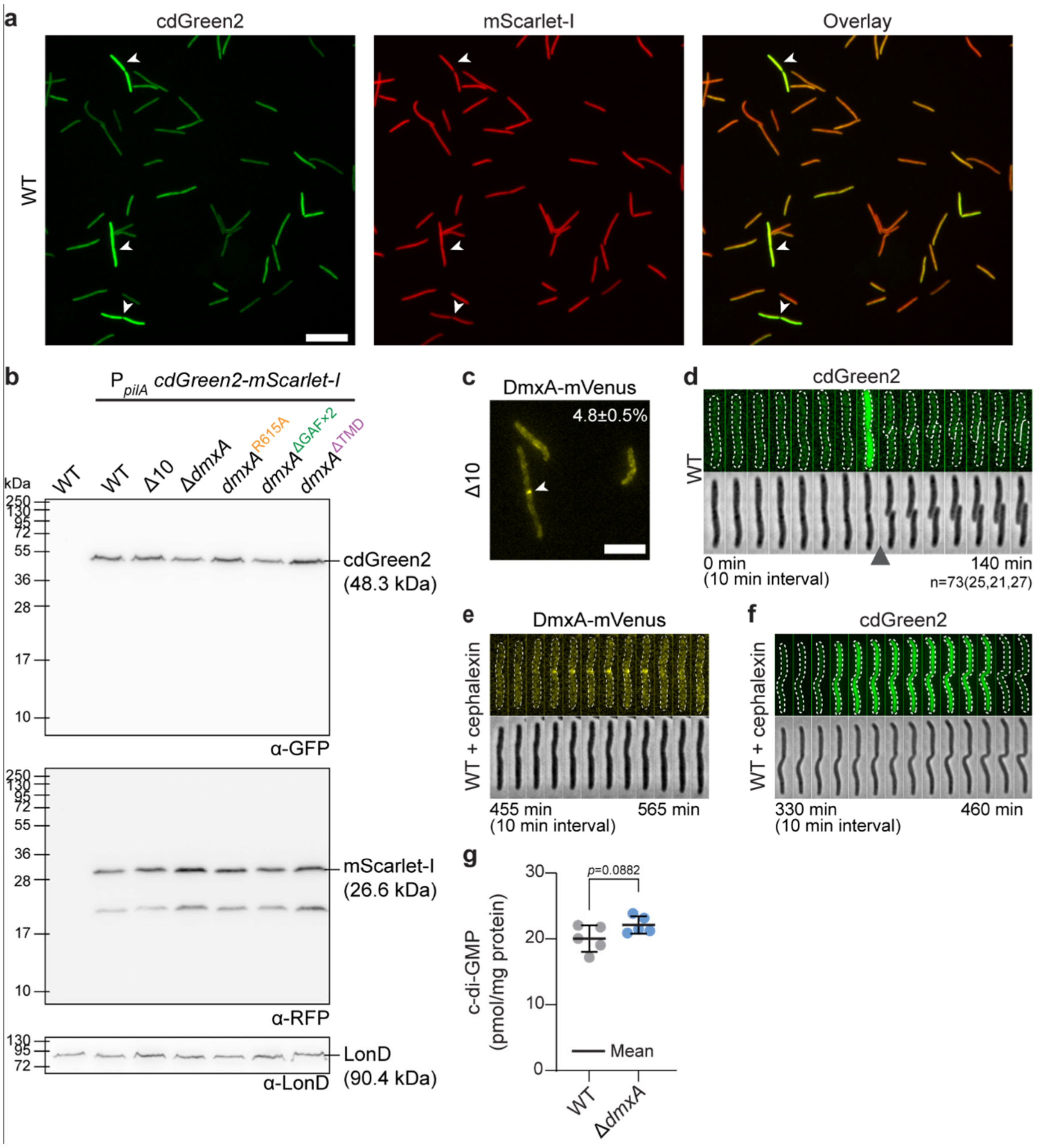

Figure S6. Accumulation and analysis of cdGreen2 and mScarlet-I fluorescence
a, Analysis of the cdGreen2 and mScarlet-I fluorescence in WT cells. White arrows indicate cells with bright cdGreen2 fluorescence. Scale bar, 10 µm. **b**, Immunoblot detection of cdGreen2 and mScarlet-I. Protein from 7.00×10^{7} cells from exponentially growing suspension cultures was loaded per lane. The same blot was stripped before applying a new antibody. LonD served as a loading control. Calculated molecular weights of proteins are indicated. Representative blots from n=2 biological replicates. **c**, Localization of the DmxA-mVenus in Δ10 cells by epifluorescence microscopy. The mean±SD percentage of cells with a cluster at mid-cell (n=600 from three biological replicates with each 200 cells) is indicated. White arrowhead indicate cluster. Scale bar, 5 µm. **d**, cdGreen2 fluorescence in WT cells during the cell cycle. Epifluorescence and phase-contrast images from time-lapse microscopy of a representative cell. In every division event, a high cdGreen2 signal was observed (n=73 division events from three biological replicates, number of events per replicate in brackets). Images were recorded every 10 min; arrowhead, completion of cytokinesis. **e-f**, Analysis of representative WT cells expressing DmxA-mVenus (e) or cdGreen2 (f) treated with cephalexin. Images were recorded every 5 min (e) or every 10 min (f). **g**, c-di-GMP level of indicated strains during growth. The level of c-di-GMP is shown as the mean±SD from n=5 biological replicates. Individual data points are shown. Samples

were compared using a two-sided Student's *t*-test. Source data are provided as a Source Data file.

Table S1. Proteins significantly enriched in DmxA-miniTurbo-FLAG proximity labeling experiments

Locus Tag	Name	Annotation	log2Ratio*	-log10(*p*-value)**
MXAN_3705	DmxA	Diguanylate cyclase	15.15	6.13
MXAN_5591		FHA domain/tetratricopeptide repeat protein	6.20	5.63
MXAN_4591	Pkn1	Serine/threonine-protein kinase	5.87	5.81
MXAN_5723		MJ0042 family finger-like domain protein	5.36	3.56
MXAN_0755	Pkn9	Serine/threonine protein kinase	5.28	3.25
MXAN_3955	Pkn10	Serine/threonine protein kinase	5.16	5.40
MXAN_4324		Uncharacterized protein	5.03	2.13
MXAN_1436		Hydrolase, alpha/beta fold family	4.95	4.13
MXAN_1131		Uncharacterized protein	4.74	3.69
MXAN_4024		Uncharacterized protein	4.64	3.48
MXAN_6266		Putative 2,3-cyclic-nucleotide 2-phosphodiesterase	4.58	2.74
MXAN_4956		Uncharacterized protein	4.45	5.56
MXAN_2680		Serine/threonine protein kinase	4.43	4.74
MXAN_1350		Histidine kinase	4.34	3.88
MXAN_5778	PilS2	Histidine kinase	4.32	4.86
MXAN_5946		Cytochrome c family protein/iron permease, FTR1 family	4.23	2.86
MXAN_4677		Uncharacterized protein	4.21	2.98
MXAN_3550		Uncharacterized protein	4.18	3.96
MXAN_7417	EpsY	OPX polysaccharide export protein	4.18	2.05
MXAN_4735		Putative membrane protein	4.16	3.20
MXAN_6605	CsdK1	PilZ-domain/DnaK family protein	4.16	4.27
MXAN_2059		Putative serine/threonine protein kinase	4.14	4.39
MXAN_1134		Adenylate/guanylate cyclase domain protein	4.12	3.31
MXAN_0117		Serine/threonine protein kinase	4.05	2.13
MXAN_4700		Serine/threonine protein kinase	4.00	3.67
MXAN_1622		Uncharacterized protein	3.98	3.40
MXAN_5710		Pyrimidine-nucleoside phosphorylase	3.86	2.38
MXAN_3108		FHA domain/tetratricopeptide repeat protein	3.79	2.47
MXAN_5321		Uncharacterized protein	3.65	4.04
MXAN_3106		Protein transporter, outer bacterial membrane secretin (Secretin) family	3.64	4.98
MXAN_2710		Putative lipoprotein	3.59	4.85
MXAN_1460	FtsK	Cell division protein	3.56	2.62
MXAN_1942		MJ0042 family finger-like domain/tetratricopeptide repeat protein	3.46	4.37
MXAN_6420	Pkn2	Serine/threonine protein kinase	3.45	2.68
MXAN_5667		Hydrolase, alpha/beta fold family	3.44	3.96
MXAN_2520		FHA domain/tetratricopeptide repeat protein	3.39	4.06
MXAN_3343		Histidine kinase	3.32	3.44
MXAN_1249		Histidine kinase	3.30	3.03
MXAN_3778	CsdK2	PilZ-domain/DnaK family protein	3.27	3.17
MXAN_4730		Lipoprotein releasing system, transmembrane protein, LolC/E family	3.25	2.87
MXAN_2683		Methyl accepting chemotaxis protein	3.23	3.02

MXAN_5813		Uncharacterized protein	3.22	4.45
MXAN_6183		Serine/threonine protein kinase	3.14	2.97
MXAN_3808		Protease 4	3.14	2.49
MXAN_3159		Uncharacterized protein	3.14	4.50
MXAN_2504		Putative general secretion pathway protein L	3.12	4.42
MXAN_3461		Oxidoreductase, short chain dehydrogenase/reductase family	3.11	2.20
MXAN_6720		Putative lipoprotein	3.08	4.70
MXAN_1313		Uncharacterized protein	3.04	3.11
MXAN_0745		Uncharacterized protein	3.03	4.34

* Fold change between the average intensities of the DmxA-miniTurbo-FLAG and the sfGFP-miniTurbo-FLAG samples.

** Log_{10} of *p*-value: Calculated using eBayes moderated *t*-statistics[29].

Table S2. Proteins significantly enriched in FtsK-miniTurbo-FLAG proximity labeling experiments

Locus Tag	Name	Annotation	log2Ratio*	-log10(*p*-value)**
MXAN_1460	FtsK	Cell division protein	10.85	6.07
MXAN_5723		MJ0042 family finger-like domain protein	6.34	3.87
MXAN_5591		FHA domain/tetratricopeptide repeat protein	6.17	6.27
MXAN_2297		Uncharacterized protein	5.95	3.90
MXAN_0960		Putative serine/threonine protein kinase	5.82	2.07
MXAN_4359	FtsH	ATP-dependent zinc metalloprotease	5.79	3.96
MXAN_3108		FHA domain/tetratricopeptide repeat protein	5.66	4.81
MXAN_4700		Serine/threonine protein kinase	5.65	4.83
MXAN_2438		Type III secretion apparatus protein, YscI/HrpB family	5.34	2.94
MXAN_2710		Putative lipoprotein	5.34	5.51
MXAN_3705	DmxA	Diguanylate cyclase	5.25	3.34
MXAN_1527		NAD dependent epimerase/dehydratase family protein	5.22	5.24
MXAN_2077		Protein kinase domain protein	5.07	3.79
MXAN_4037		Cytidylate kinase	4.98	3.80
MXAN_3106		Protein transporter, outer bacterial membrane secretin (Secretin) family	4.90	5.09
MXAN_0363	TfcP	T4P motility protein	4.80	5.48
MXAN_6605	CsdK1	PilZ-domain/DnaK family protein	4.78	4.31
MXAN_6860	AglS	Gliding motility protein	4.78	2.82
MXAN_1419		Uncharacterized protein	4.77	3.92
MXAN_4186		Uncharacterized protein	4.71	3.46
MXAN_5765		Putative general secretion pathway protein A	4.69	4.47
MXAN_6224		DNA-binding response regulator, Fis family	4.68	4.14
MXAN_5772	PilQ	T4P secretin	4.66	3.77
MXAN_5028		Putative membrane protein	4.59	4.64
MXAN_3001	TsaP	T4P motility protein	4.58	6.12
MXAN_0755	Pkn9	Serine/threonine protein kinase	4.57	2.84
MXAN_4024		Uncharacterized protein	4.51	3.17
MXAN_5075		Ribose-phosphate pyrophosphokinase	4.49	3.01
MXAN_5319	PglC	Tetratricopeptide repeat protein	4.43	5.96
MXAN_6705	PilT5	T4P retraction ATPase	4.41	4.04
MXAN_3013		ATP-dependent protease ATPase subunit HslU	4.37	5.57
MXAN_4667		General secretion pathway protein E, N-terminal domain protein	4.32	4.10
MXAN_6183		Serine/threonine protein kinase	4.29	3.94
MXAN_6861	AglQ	Gliding motility protein	4.26	3.75
MXAN_4737		HDIG domain protein	4.17	3.51
MXAN_2968		Efflux transporter, RND family, MFP subunit	4.12	5.30
MXAN_6420	Pkn2	Serine/threonine protein kinase	4.11	4.07
MXAN_3202	Pkn3	Serine/threonine protein kinase	4.05	5.55
MXAN_1361		FHA domain protein	4.04	2.87

MXAN_2312		Uncharacterized protein	4.04	3.42
MXAN_1350		Histidine kinase	4.02	5.82
MXAN_0459		Histidine kinase	3.98	4.29
MXAN_3337		NAD-dependent protein deacylase	3.95	3.38
MXAN_5597	FtsZ	Cell division protein	3.93	3.86
MXAN_3550		Uncharacterized protein	3.91	5.69
MXAN_2483		Type II DNA topoisomerase, B subunit	3.91	4.74
MXAN_7208		Serine/threonine protein kinase	3.90	4.99
MXAN_2480		Signal peptide peptidase SppA, 36K type	3.89	3.75
MXAN_2550	Pkn6	Serine/threonine protein kinase	3.85	2.98
MXAN_3710		Serine/threonine protein kinase	3.83	2.02
MXAN_5152		OmpA family protein	3.83	5.40
MXAN_5735	FtsY	Signal recognition particle receptor	3.79	5.32
MXAN_2680		Serine/threonine protein kinase	3.79	4.07
MXAN_1178		FKBP-type peptidyl-prolyl isomerase, trigger factor family	3.78	2.91
MXAN_5783	PilA	T4P major pilin	3.77	5.24
MXAN_7025		DnaK family protein	3.76	3.88
MXAN_4371		Serine/threonine protein kinase	3.73	3.44
MXAN_2586		Serine/threonine protein kinase	3.72	5.00
MXAN_0625		Aldehyde dehydrogenase	3.69	2.37
MXAN_1948		TPR domain protein	3.65	5.25
MXAN_5786	PilC	T4P motility protein	3.57	3.67
MXAN_7371		Serine/threonine protein kinase	3.54	3.43
MXAN_5129		Uncharacterized protein	3.52	4.58
MXAN_4094		Uncharacterized protein	3.48	3.27
MXAN_0018		Serine/threonine protein kinase	3.48	3.78
MXAN_6704		Acetyltransferase, GNAT family	3.40	3.37
MXAN_0882		Serine/threonine protein kinase	3.37	3.68
MXAN_3570		Uncharacterized protein	3.37	4.62
MXAN_4735		Putative membrane protein	3.36	4.36
MXAN_1984		Uncharacterized protein	3.34	4.76
MXAN_0653		Peptidase, S8A (Subtilisin) subfamily	3.33	3.38
MXAN_6310		AhpC/TSA family protein	3.33	2.08
MXAN_2444		Uncharacterized protein	3.27	2.86
MXAN_1995	PilT	T4P retraction ATPase	3.27	3.01
MXAN_2512	GspF	General secretion pathway protein F	3.25	3.15
MXAN_6570		Serine/threonine protein kinase	3.22	2.82
MXAN_0344		Metallophosphoesterase	3.16	3.89
MXAN_6155		Alkylphosphonate utilization operon protein PhnA	3.13	5.22
MXAN_0871		Putative serine/threonine protein kinase	3.13	2.30
MXAN_5696		Serine/threonine protein kinase	3.13	3.87
MXAN_4591	Pkn1	Serine/threonine protein kinase	3.12	3.72
MXAN_6189		Uncharacterized protein	3.12	3.63
MXAN_4867	GltF	Gliding motility protein	3.12	4.51

MXAN_6611		Uncharacterized protein	3.11	3.34
MXAN_2013		Trigger factor	3.06	5.01
MXAN_0599		Uncharacterized protein	3.06	2.33
MXAN_5824		Putative membrane protein	3.06	3.67
MXAN_3984		SPFH/band 7 domain protein	3.04	2.40
MXAN_3918		Glutamate synthase, small subunit	3.04	3.80
MXAN_6751		Uncharacterized protein	3.03	4.12
MXAN_2156		Serine/threonine protein kinase	3.03	3.98

* Fold change between the average intensities of the FtsK-miniTurbo-FLAG and the sfGFP-miniTurbo-FLAG samples.

** Log_{10} of *p*-value: Calculated using eBayes moderated *t*-statistics[29].

Table S3. Strains used in this work

Strain	Genotype	Reference
M. xanthus		
DK1622	Wildtype	[30]
DK10410	Δ*pilA*	[31]
SA3922	Δ*gltB*	[11]
SA8802	Δ*frzE*	[6]
SA3387	Δ*mglB*	[4]
SA7442	Δ*dmxA*	This study
SA7447	Δ*dmxA attB::*pTP140 (P_{nat} *dmxA*)	This study
SA7466	Δ*dmxA* Δ*pilA*	This study
SA12010	Δ*dmxA* Δ*gltB*	This study
SA7479	Δ*dmxA* Δ*frzE*	This study
SA7485	*dmxA-mVenus*	This study
SA8589	*dmxA-mVenus* Δ*gltB*	This study
SA8580	*dmxA*^{E626A}*-mVenus*	This study
SA7488	*dmxA*^{R615A}*-mVenus*	This study
SA12015	*dmxA*$^{\Delta GAF\times2\ (\Delta 223\text{-}532)}$*-mVenus*	This study
SA12034	*dmxA*$^{\Delta TMD\ (\Delta 3\text{-}222)}$*-mVenus*	This study
SA12065	*dmxA*$^{TMD\ (1\text{-}167)}$*-mVenus*	This study
SA6755	Δ*ftsZ MXAN_18-19::*pMAT86 (P_{van} *ftsZ*)	This study
SA8511	Δ*ftsZ MXAN_18-19::*pMAT86 (P_{van} *ftsZ*) *dmxA-mVenus*	This study
SA8596	Δ*dmxA attB::*pMP172 (P_{pilA} *dmxA-miniTurbo-FLAG*)	This study
SA12038	*attB::*pMH110 (P_{pilA} *ftsK-miniTurbo-FLAG*)	This study
SA12027	WT *MXAN_18-19::*pMH97 (P_{van} *sfGFP-miniTurbo-FLAG*)	[32]
SA9082	*attB::*pMH123 (P_{pilA} *cdGreen2 mScarlet-I*)	This study
SA12093	Δ*gltB attB::*pMH123 (P_{pilA} *cdGreen2 mScarlet-I*)	This study
SA12091	Δ*dmxA attB::*pMH123 (P_{pilA} *cdGreen2 mScarlet-I*)	This study
SA12092	Δ*dmxA* Δ*gltB attB::*pMH123 (P_{pilA} *cdGreen2 mScarlet-I*)	This study
SA5666	Δ10 (Δ*MXAN_1525*, Δ*MXAN_4029*, Δ*MXAN_5199*, Δ*MXAN_7362,* Δ*gacB* (Δ*MXAN_4463*), Δ*gacA* (Δ*MXAN_2643*), Δ*MXAN_2997*, Δ*MXAN_5366*, Δ*dmxB* (Δ*MXAN_3735*), Δ*MXAN_5791*)	This study
SA12098	Δ10* *dmxA-mVenus*	This study
SA9083	Δ10* *attB::*pMH123 (P_{pilA} *cdGreen2 mScarlet-I*)	This study
SA12063	Δ10* Δ*gltB*	This study
SA9084	Δ10* Δ*gltB attB::*pMH123 (P_{pilA} *cdGreen2 mScarlet-I*)	This study
SA13309	*dmxA*^{R615A}	This study
SA13310	*dmxA*^{R615A} *attB::*pMH123 (P_{pilA} *cdGreen2 mScarlet-I*)	This study
SA12023	*dmxA*$^{\Delta GAF\times2\ (\Delta 223\text{-}532)}$	This study
SA12097	*dmxA*$^{\Delta GAF\times2\ (\Delta 223\text{-}532)}$ *attB::*pMH123 (P_{pilA} *cdGreen2 mScarlet-I*)	This study
SA12029	*dmxA*$^{\Delta TMD\ (\Delta 3\text{-}222)}$	This study
SA12095	*dmxA*$^{\Delta TMD\ (\Delta 3\text{-}222)}$ *attB::*pMH123 (P_{pilA} *cdGreen2 mScarlet-I*)	This study
SA7192	*pilQ-sfGFP*	[33]
SA12017	*pilQ-sfGFP* Δ*gltB*	[25]
SA8505	Δ*dmxA pilQ-sfGFP*	This study
SA12019	Δ*dmxA pilQ-sfGFP* Δ*gltB*	This study
SA7896	*mCherry-pilM*	[22]
SA8576	Δ*dmxA mCherry-pilM*	This study

SA9307	*mCherry-pilT*	[34]
SA8575	Δ*dmxA mCherry-pilT*	This study
SA9300	*pilB-mCherry*	[34]
SA8572	Δ*dmxA pilB-mCherry*	This study
SA7507	*romR-mCherry*	[6]
SA12056	*romR-mCherry* Δ*gltB*	This study
SA7459	Δ*dmxA romR-mCherry*	This study
SA12064	Δ*dmxA romR-mCherry* Δ*gltB*	This study
SA10043	*mglB-mVenus*	[7]
SA12024	Δ*dmxA mglB-mVenus*	This study
SA8185	*mglA-mVenus*	[6]
SA7480	Δ*dmxA mglA-mVenus*	This study
SA7195	*sgmX-mVenus*	[33]
SA12014	Δ*dmxA sgmX-mVenus*	This study
E. coli		
Mach1	Δ*recA*1398 *endA*1 *tonA* Φ80Δ*lacM*15 Δ*lacX*74 *hsdR*(r_K^- m_K^+)	Invitrogen
Rosetta 2 (DE3)	F^- *ompT hsdS*$_B$(r_B^- m_B^-) *gal dcm* (DE3) pRARE2	Novagen/Merck

* Δ10 mutations are described in detail in strain SA5666.

Table S4. Plasmids used in this work.

Plasmid	Description	Reference
pBJ114	*galK,* Km^r	[35]
pSW105	*attP,* P_{pilA} Km^r	[36]
pSWU30	*attP,* Tet^r	[37]
pMR3691	*MXAN_18-19, vanR* P_{van}, Tet^r	[38]
pMAL-c6t	Expression vector, P_{tac}, His_6-MalE, Amp^r	NEB
pET28(+)	Expression vector, P_{T7} His_6, Km^r	Novagen
pMAT162	pBJ114, in-frame deletion construct for *pilA,* Km^r	[6]
pDK25	pBJ114, in-frame deletion construct for *gltB,* Km^r	[11]
pAP19	pBJ114, in-frame deletion construct for *frzE,* Km^r	[33]
pMP072	pBJ114, in-frame deletion construct for *dmxA,* Km^r	This work
pTP140	pSWU30, P_{nat} *dmxA,* Tet^r	[39]
pMP092	pSWU30, P_{nat} *dmxA-mVenus,* Tet^r	This work
pMP093	pBJ114, *dmxA* replacement by *dmxA-mVenus*, Km^r	This work
pMP164	pBJ114, for native site integration of *dmxA-mVenus,* Km^r	This work
pMP165	pBJ114, for native site integration of *dmxA*^{E626A}*-mVenus,* Km^r	This work
pMP095	pBJ114, *dmxA* replacement by *dmxA*^{R615A}*-mVenus,* Km^r	This work
pKH02	pBJ114, in-frame deletion of *mVenus* in the *dmxA*^{R615A}*-mVenus* strain, Km^r	This work
pMP175	pBJ114, *dmxA* or *dmxA-mVenus* replacement by *dmxA*$^{\Delta GAF\times 2\ (\Delta 223\text{-}532)}$, Km^r	This work
pMP179	pBJ114, *dmxA* or *dmxA-mVenus* replacement by *dmxA*$^{\Delta TMD\ (\Delta 3\text{-}222)}$, Km^r	This work
pMP182	pBJ114, for native site integration of *dmxA*$^{TMD\ (1\text{-}167)}$*-mVenus,* Km^r	This work
pMH113	pMAL-c6t, *malE-dmxA*$^{WT(223\text{-}722)}$, Amp^r	This work
pTP139	pET28a, His_6-*dmxA*$^{223\text{-}722,\ E626A}$, Km^r	This work
pMH117	pMAL-c6t, *malE-dmxA*$^{E626A\ (223\text{-}722,\ E626A)}$, Amp^r	This work
pMP082	pET28a, His_6-*dmxA*$^{223\text{-}722,\ R615A}$, Km^r	This work
pMH116	pMAL-c6t, *malE-dmxA*$^{R615A\ (223\text{-}722,\ R615A)}$, Amp^r	This work
pMH114	pMAL-c6t, *malE-dmxA*$^{GAF\times 2\ (223\text{-}547)}$, Amp^r	This work
pMH115	pMAL-c6t, *malE-dmxA*$^{GGDEF\ (546\text{-}722)}$, Amp^r	This work
pMAT74	pBJ114, in-frame deletion construct for *ftsZ,* Km^r	This work
pMAT86	pMR3691, *ftsZ*, Tet^r	This work
pMH52	pEX-k168, *miniTurbo-FLAG*, Km^r	This work
pMP172	pSW105, *dmxA-miniTurbo-FLAG* , Km^r	This work
pMH98	pBJ114, *ftsK* replacement by *ftsK-miniTurbo-FLAG*, Km^r	This work
pMH110	pSW105, *ftsK-miniTurbo-FLAG*, Km^r	This work

pBBR15.2-2H12.D11opt-scarRef	pBBR15.2, $P_{tet\text{-}const}$ *cdGreen2 mScarlet-I*, Gm^r	40
pMH123	pSW105, *cdGreen2 mScarlet-I*, Km^r	This work
pAP37	pBJ114, *pilQ* replacement by *pilQ-sfGFP*, Km^r	33
pMAT336	pBJ114, *pilM* replacement by *mCherry-pilM*, Km^r	22
pMEM33	pBJ114, *pilT* replacement by *mCherry-pilT*, Km^r	34
pMEM23	pBJ114, *pilB* replacement by *pilB-mCherry*, Km^r	34
pLC32	pBJ114, *romR* replacement by *romR-mCherry*, Km^r	6
pLC58	pBJ114, *mglB* replacement by *mglB-mVenus*, Km^r	7
pLC20	pBJ114, *mglA* replacement by *mglA-mVenus*, Km^r	6
pAP35	pBJ114, *sgmX* replacement by *sgmX-mVenus*, Km^r	33
pDJS01	pBJ114, in-frame deletion construct for *dmxB* (*MXAN_3735*), Km^r	39
pDJS02	pBJ114, in-frame deletion construct for *MXAN_5366*, Km^r	39
pDJS03	pBJ114, in-frame deletion construct for *MXAN_7362*, Km^r	39
pIH01	pBJ114, in-frame deletion construct for *gacA* (*MXAN_4463*), Km^r	39
pIH03	pBJ114, in-frame deletion construct for *MXAN_5791*, Km^r	39
pNGS010	pBJ114, in-frame deletion construct for *MXAN_2997*, Km^r	39
pTP120	pBJ114, in-frame deletion construct for *MXAN_1525*, Km^r	39
pTP121	pBJ114, in-frame deletion construct for *MXAN_5199*, Km^r	39
pTP125	pBJ114, in-frame deletion construct for *gacB* (*MXAN_2643*), Km^r	39
pTP127	pBJ114, in-frame deletion construct for *MXAN_4029*, Km^r	39

Table S5. Oligonucleotides used in this work[1]

Primer name	Sequence 5'-3'		Brief description
3702_map_fwd2	TGACCACCTGGTCTGTACGC	Operon mapping Fragment A	Primers used for operon mapping.
3703_map_rev2	GGGGGCCATAGTCCTTCTGG		
3704_map_rev2	TCATTGCGCAGGGCCTCATT	Operon mapping Fragment B	
3703_map_fwd2	CGCGAACCAGGCGATGATTG		
3704_map_fwd2	AGGCCCTGCGCAATGAGATT	Operon mapping Fragment C	
3705_map_rev2	AATGGCCACCACGACGAAGG		
3705_map_fwd2	GCCGCTGAGGATCACCATGT	Operon mapping Fragment D	
3706_map_rev2	CTCTGGGGCATCGGCCTG		
ftsZ-up EcoRI	GCGCGAATTCGGTGGACACGGATGGCGACG		For deletion of *ftsZ*
ftsZ-overlapping reverse	CGTCTGGCCCTTGTTCTGATCGAACTGGTC		
ftsZ-overlapping forward	CAGAACAAGGGCCAGACGGAACTGCCGTAA		
ftsZ-down HindIII	GCGCAAGCTTTTGCGCAGCTGCCAGCCGATG		
ftsZ-start NdeI	GGAATTCCATATGGACCAGTTCGATCAGAAC		For expression of *ftsZ* under the P_{van} promoter
ftsZ-stop KpnI	GCGCGGTACCTTACGGCAGTTCCGTCTGGC		
sfGFP_fwd_NdeI	GCGCCATATGAGCAAAGGAGAAGAACT		For expression of *sfGFP-miniTurbo-FLAG* under the P_{van} promoter
sfGFP_rev_OV	CGCCCCCGCCTTTGTAGAGCTCATCCATGC		
TurboID_fwd_OV	GCTCTACAAAGGCGGGGGCGGGAGCATGAT		
TurboID_rev_EcoRI	GCGCGAATTCTCACTTGTCGTCGTCGTC		
FtsK_A_BamHI	GCGCGGATCCACGAGGACGACATGCTCGACGC		For replacement of *ftsK* with *ftsK-miniTurbo-FLAG*
FtsK_B_TID_OV	CGCCCCCGCCCATGGCCCCGGCGCCGGGCA		
TID_fwd_FtsK_OV	CGGGGCCATGGGCGGGGGCGGGAGCATGAT		
TID_rev_FtsK_OV	CACGTGGGCCTCACTTGTCGTCGTCGTCCT		
FtsK_C_TID_OV	CGACAAGTGAGGCCCACGTGGCCGCCGTCT		
FtsK_D_HindIII	GCGCAAGCTTCACCTTGAGGGCCACGAAGCCGG		
FtsK_fwd_XbaI_2	GCGCTCTAGACAGCCGAAATGTAGGTTCCCCGTCCTGAGC		For expression of *ftsK-miniTurbo-FLAG* from the *attB* site and P_{pilA}
FtsK_B_TID_OV 2	CGCCCCCGCCCATGGCCCCGGCGCCGGGCATGTCG		
TID_fwd_FtsK_OV	CGGGGCCATGGGCGGGGGCGGGAGCATGAT		
Flag_rev HindIII	GCGCAAGCTTTCACTTGTCGTCGTCGTC		
MalE-fwd_NotI	GCGCGCGGCCGCGAGATCGAAGGCGCCGTGC		For expression and purification of MalE-tagged DmxA variants
DmxA_Rev_HindIII	GCGCAAGCTTTCAGGACGCGTTCGCCGCCT		
DmxA_GAF2_rev_HindIII	CTGAGGATCCTCACGTGGTGGCCATGCGCTCCA		
DmxA_GGDEFonly_Fwd_NotI	CTGAGGATCCTCACGTGGTGGCCATGCGCTCCA		
3705 GAF1 forw NdeI	ATCGCATATGGAGATCGAAGGCGCCGTGC		For expression and purification of His6-tagged DmxA protein variants
3705 E626A (-)	GACGAACTCCGCGCCGCCGTA		
3705 E626A (+)	TACGGCGGCGCGGAGTTCGTC		
3705 R615A (-)	GTCCGTATCGGCCGCCATCGTC		
3705 R615A (+)	GACGATGGCGGCCGATACGGAC		
3705 rev BamHI	ATCGGGATCCTCAGGACGCGTTCGCCGC		
cdG-Sensor_fwd_XbaI	ATGCTCTAGAATGAATTCGGAACCGCCGCC		For expression of cdGreen and
cdG-Sensor_rev_HindIII	ATTCAAGCTTTCACTTGTACAGTTCATCCATACCACCG		

		mScarlet-I from the *attB* site and the P_{pilA}
3705_A	ATCGGGTACCAAGGTGACGTCGCACAAGAT	For deletion of *dmxA*
3705_B	CTTCAGCTGAGACGGAAACTCGGGAAG	
3705_C	TTTCCGTCTCAGCTGAAGGCGGCGAAC	
3705_D	ATCGTCTAGACATCACCGGGTGGACCGTCA	
3704 prmt forw +XbaI	ATCGTCTAGATTCCTCAACGCGCTGGCGCTG	For P_{nat} *dmxA-mVenus*
3705_rev no stop 1	GCCGCCGCCGGACGCGTTCGCCGCCTTCAG	
3705_mVenus fw	AACGCGTCCGGCGGCGGCGGCTCCATGGTGAGCAAGGG	
mVenus_Kpn rev	CGCGCCGGGTACCTTACTTGTACAGCTCGTCCA	
3705_native forw	ATATGGTACCTCCGCTACGGGGCGCCGCTG	For replacement of *dmxA* with *dmxA-mVenus*
3705_native middle rev	CCCGGCTGCATCAAGGACTTACTTGTACAGCTCGTC	
3705_native middle fw	GACGAGCTGTACAAGTAAGTCCTTGATGCAGCCGGG	
3705_native rev	CGCGTCTAGACATCCCCGAGTCGGCCATCG	
3705_PpilA forw	CGCGTCTAGAATGACGTCGCTTCCCGAGTT	For P_{pilA} *dmxA-miniTurbo-FLAG*
3705_miniTurboID fw	AACGCGTCCGGCGGCGGCGGCTCCATGATCCCGCTCCTGA	
3705_B (GAFx1)	CGCGCGCAGCTTCACCGAGGCCACCAG	For in-frame deletion of the GAF domains of *dmxA*
3705_C (GAFx2)	TCGGTGAAGCTGCGCGCGCAGCTCTAC	
3705_D (GAFx2)	ATCGGGTACCGGACGCGTTCGCCGCCTTCA	
3705_B3 (TMD)	TTCGATCTCCGTCATGGGGACTCCAGG	For native site integration of $dmxA^{\Delta TMD}$ *-mVenus*
3705_C3 (TMD)	CCCATGACGGAGATCGAAGGCGCCGTG	
3705_D (TMD)2	ATCGTCTAGACAGCGGCGCCCCGTAGCGGA	
3705_TMH rev	GGAGCCGCCGCCGCCCAGCACCAGGTGGTAGAGGC	For native site integration of $dmxA^{TMD}$ $^{(1\text{-}167)}$*-mVenus*
3705_mVenus fw2	CTGGTGCTGGGCGGCGGCGGCTCCATGGTGAGCAAGGG	
3705_del_mVenus_(-)	AAGGACTCAGGACGCGTTCGCCGCCTTCAG	For deletion of *mVenus*
3075_del_mVenus_(+)	AACGCGTCCTGAGTCCTTGATGCAGCCGGG	

[1] Underlined sequences indicate restriction sites.

Table S6. Fully sequenced myxobacterial genomes used for the 16S rRNA tree

Species and strain name
Anaeromyxobacter dehalogenans 2CP-C
Anaeromyxobacter sp. Fw109-5
Anaeromyxobacter sp. K
Anaeromyxobacter oryzae Red232
Anaeromyxobacter paludicola Red630
Archangium gephyra DSM 2261
Archangium violaceum SDU34
Chondromyces crocatus Cm c5
Corallococcus macrosporus DSM 14697
Corallococcus coralloides DSM 2259
Cystobacter fuscus DSM 52655
Haliangium ochraceum DSM 14365
Labilithrix luteola DSM 27648
Melittangium boletus DSM 14713
Minicystis rosea DSM 24000
Myxococcus fulvus 124B02
Myxococcus hansupus (*Myxococcus* sp. mixupus)
Myxococcus stipitatus DSM 14675
Myxococcus xanthus DK1622
Nannocystis sp. fl3
Polyangium aurulentum SDU3-1
Sandaracinus amylolyticus DSM 53668
Sorangium cellulosum So ce 56
Stigmatella aurantiaca DW4/3-1
Vulgatibacter incomptus DSM 27710

Supplementary References

1. Black, W.P., Xu, Q. & Yang, Z. Type IV pili function upstream of the Dif chemotaxis pathway in *Myxococcus xanthus* EPS regulation. *Mol Microbiol* **61**, 447-456 (2006).

2. Carreira, L.A.M., Szadkowski, D., Lometto, S., Hochberg, G.K.A. & Søgaard-Andersen, L. Molecular basis and design principles of switchable front-rear polarity and directional migration in *Myxococcus xanthus*. *Nat Commun* **14**, 4056 (2023).

3. Carreira, L.A.M., Tostevin, F., Gerland, U. & Søgaard-Andersen, L. Protein-protein interaction network controlling establishment and maintenance of switchable cell polarity. *PLOS Genet* **16**, e1008877 (2020).

4. Leonardy, S. *et al.* Regulation of dynamic polarity switching in bacteria by a Ras-like G-protein and its cognate GAP. *EMBO J* **29**, 2276-2289 (2010).

5. Zhang, Y., Franco, M., Ducret, A. & Mignot, T. A bacterial Ras-like small GTP-binding protein and its cognate GAP establish a dynamic spatial polarity axis to control directed motility. *PLoS Biol* **8**, e1000430 (2010).

6. Szadkowski, D. *et al.* Spatial control of the GTPase MglA by localized RomR-RomX GEF and MglB GAP activities enables *Myxococcus xanthus* motility. *Nat Microbiol* **4**, 1344-1355 (2019).

7. Szadkowski, D., Carreira, L.A.M. & Søgaard-Andersen, L. A bipartite, low-affinity roadblock domain-containing GAP complex regulates bacterial front-rear polarity. *PLOS Genet* **18**, e1010384 (2022).

8. Leonardy, S., Freymark, G., Hebener, S., Ellehauge, E. & Søgaard-Andersen, L. Coupling of protein localization and cell movements by a dynamically localized response regulator in *Myxococcus xanthus*. *EMBO J* **26**, 4433-4444 (2007).

9. Keilberg, D., Wuichet, K., Drescher, F. & Søgaard-Andersen, L. A response regulator interfaces between the Frz chemosensory system and the MglA/MglB GTPase/GAP module to regulate polarity in *Myxococcus xanthus*. *PLOS Genet* **8**, e1002951 (2012).

10. Zhang, Y., Guzzo, M., Ducret, A., Li, Y.Z. & Mignot, T. A dynamic response regulator protein modulates G-protein-dependent polarity in the bacterium *Myxococcus xanthus*. *PLOS Genet* **8**, e1002872 (2012).

11. Jakobczak, B., Keilberg, D., Wuichet, K. & Søgaard-Andersen, L. Contact- and protein transfer-dependent stimulation of assembly of the gliding motility machinery in *Myxococcus xanthus*. *PLOS Genet* **11**, e1005341 (2015).

12. Luciano, J. *et al.* Emergence and modular evolution of a novel motility machinery in bacteria. *PLOS Genet* **7**, e1002268 (2011).

13. Mignot, T., Shaevitz, J.W., Hartzell, P.L. & Zusman, D.R. Evidence that focal adhesion complexes power bacterial gliding motility. *Science* **315**, 853-856 (2007).

14. Nan, B. *et al.* Flagella stator homologs function as motors for myxobacterial gliding motility by moving in helical trajectories. *Proc Natl Acad Sci USA* **110**, E1508-1513 (2013).

15. Nan, B., Mauriello, E.M., Sun, I.H., Wong, A. & Zusman, D.R. A multi-protein complex from *Myxococcus xanthus* required for bacterial gliding motility. *Mol Microbiol* **76**, 1539-1554 (2010).

16. Wartel, M. *et al.* A versatile class of cell surface directional motors gives rise to gliding motility and sporulation in *Myxococcus xanthus*. *PLOS Biol* **11**, e1001728 (2013).

17. Treuner-Lange, A. *et al.* The small G-protein MglA connects to the MreB actin cytoskeleton at bacterial focal adhesions. *J Cell Biol* **210**, 243-256 (2015).

18. Islam, S.T. *et al.* Unmasking of the von Willebrand A-domain surface adhesin CglB at bacterial focal adhesions mediates myxobacterial gliding motility. *Sci Adv* **9**, eabq0619 (2023).

19. Nan, B. *et al.* Myxobacteria gliding motility requires cytoskeleton rotation powered by proton motive force. *Proc Natl Acad Sci USA* **108**, 2498-2503 (2011).

20. Sun, M., Wartel, M., Cascales, E., Shaevitz, J.W. & Mignot, T. Motor-driven intracellular transport powers bacterial gliding motility. *Proc Natl Acad Sci USA* **108**, 7559-7564 (2011).

21. Chang, Y.W. *et al.* Architecture of the type IVa pilus machine. *Science* **351**, aad2001 (2016).

22. Treuner-Lange, A. *et al.* PilY1 and minor pilins form a complex priming the type IVa pilus in *Myxococcus xanthus*. *Nat Commun* **11**, 5054 (2020).

23. Nudleman, E., Wall, D. & Kaiser, D. Polar assembly of the type IV pilus secretin in *Myxococcus xanthus*. *Mol Microbiol* **60**, 16-29 (2006).

24. Friedrich, C., Bulyha, I. & Søgaard-Andersen, L. Outside-in assembly pathway of the type IV pilus system in *Myxococcus xanthus*. *J Bacteriol* **196**, 378-390 (2014).

25. Herfurth, M., Pérez-Burgos, M. & Søgaard-Andersen, L. The mechanism for polar localization of the type IVa pilus machine in *Myxococcus xanthus*. *mBio*, in press (2023).

26. Bischof, L.F., Friedrich, C., Harms, A., Søgaard-Andersen, L. & van der Does, C. The type IV pilus assembly ATPase PilB of *Myxococcus xanthus* interacts with the inner membrane platform protein PilC and the nucleotide-binding protein PilM. *J Biol Chem* **291**, 6946-6957 (2016).

27. Takhar, H.K., Kemp, K., Kim, M., Howell, P.L. & Burrows, L.L. The platform protein is essential for type IV pilus biogenesis. *J Biol Chem* **288**, 9721-9728 (2013).

28. Kuzmich, S. *et al.* CRP-like transcriptional regulator MrpC curbs c-di-GMP and 3',3'-cGAMP nucleotide levels during development in *Myxococcus xanthus*. *mBio* **13**, e0004422 (2021).

29. Smyth, G.K. Linear models and empirical Bayes methods for assessing differential expression in microarray experiments. *Stat Appl Genet Mol Biol* **3**, Article3 (2004).

30. Kaiser, D. Social gliding is correlated with the presence of pili in *Myxococcus xanthus*. *Proc Natl Acad Sci USA* **76**, 5952-5956 (1979).

31. Wu, S.S., Wu, J. & Kaiser, D. The *Myxococcus xanthus pilT* locus is required for social gliding motility although pili are still produced. *Mol Microbiol* **23**, 109-121 (1997).

32. Herfurth, M., Müller, F., Søgaard-Andersen, L. & Glatter, T. A miniTurbo-based proximity labeling protocol to identify conditional protein interactomes *in vivo* in *Myxococcus xanthus*. *STAR Protoc* **4**, 102657 (2023).

33. Potapova, A., Carreira, L.A.M. & Søgaard-Andersen, L. The small GTPase MglA together with the TPR domain protein SgmX stimulates type IV pili formation in *M. xanthus*. *Proc Natl Acad Sci USA* **117**, 23859-23868 (2020).

34. Oklitschek, M., Carreira, L.A.M., Muratoğlu, M., Søgaard-Andersen, L. & Treuner-Lange, A. Combinatorial control of type IVa pili formation by the four polarized regulators MglA, SgmX, FrzS and SopA. *bioRxiv*, 2024.2003.2011.584430 (2024).

35. Julien, B., Kaiser, A.D. & Garza, A. Spatial control of cell differentiation in *Myxococcus xanthus*. *Proc Natl Acad Sci USA* **97**, 9098-9103 (2000).

36. Jakovljevic, V., Leonardy, S., Hoppert, M. & Søgaard-Andersen, L. PilB and PilT are ATPases acting antagonistically in type IV pilus function in *Myxococcus xanthus*. *J Bacteriol* **190**, 2411-2421 (2008).

37. Wu, S.S. & Kaiser, D. Regulation of expression of the *pilA* gene in *Myxococcus xanthus*. *J Bacteriol* **179**, 7748-7758 (1997).

38. Iniesta, A.A., Garcia-Heras, F., Abellon-Ruiz, J., Gallego-Garcia, A. & Elias-Arnanz, M. Two systems for conditional gene expression in *Myxococcus xanthus* inducible by isopropyl-beta-D-thiogalactopyranoside or vanillate. *J Bacteriol* **194**, 5875-5885 (2012).

39. Skotnicka, D. *et al.* C-di-GMP regulates type IV pili-dependent-motility in *Myxococcus xanthus*. *J Bacteriol* **198**, 77-90 (2015).

40. Kaczmarczyk, A. *et al.* A genetically encoded biosensor to monitor dynamic changes of c-di-GMP with high temporal resolution. *Nat Commun* **15**, 3920 (2024).
